# MOCCA: a flexible suite for modelling DNA sequence motif occurrence combinatorics

**DOI:** 10.1186/s12859-021-04143-2

**Published:** 2021-05-07

**Authors:** Bjørn André Bredesen, Marc Rehmsmeier

**Affiliations:** 1 Computational Biology Unit, Department of Informatics, University of Bergen, P.O. Box 7803, 5020 Bergen, Norway; 2 Department of Biology, Humboldt-Universität zu Berlin, Unter den Linden 6, 10099 Berlin, Germany

**Keywords:** *Cis*-regulatory element, Motif, Machine learning, Support vector machine, Random forest

## Abstract

**Background:**

*Cis*-regulatory elements (CREs) are DNA sequence segments that regulate gene expression. Among CREs are promoters, enhancers, Boundary Elements (BEs) and Polycomb Response Elements (PREs), all of which are enriched in specific sequence motifs that form particular occurrence landscapes. We have recently introduced a hierarchical machine learning approach (SVM-MOCCA) in which Support Vector Machines (SVMs) are applied on the level of individual motif occurrences, modelling local sequence composition, and then combined for the prediction of whole regulatory elements. We used SVM-MOCCA to predict PREs in *Drosophila* and found that it was superior to other methods. However, we did not publish a polished implementation of SVM-MOCCA, which can be useful for other researchers, and we only tested SVM-MOCCA with IUPAC motifs and PREs.

**Results:**

We here present an expanded suite for modelling CRE sequences in terms of motif occurrence combinatorics—Motif Occurrence Combinatorics Classification Algorithms (MOCCA). MOCCA contains efficient implementations of several modelling methods, including SVM-MOCCA, and a new method, RF-MOCCA, a Random Forest–derivative of SVM-MOCCA. We used SVM-MOCCA and RF-MOCCA to model *Drosophila* PREs and BEs in cross-validation experiments, making this the first study to model PREs with Random Forests and the first study that applies the hierarchical MOCCA approach to the prediction of BEs. Both models significantly improve generalization to PREs and boundary elements beyond that of previous methods—including 4-spectrum and motif occurrence frequency Support Vector Machines and Random Forests—, with RF-MOCCA yielding the best results.

**Conclusion:**

MOCCA is a flexible and powerful suite of tools for the motif-based modelling of CRE sequences in terms of motif composition. MOCCA can be applied to any new CRE modelling problems where motifs have been identified. MOCCA supports IUPAC and Position Weight Matrix (PWM) motifs. For ease of use, MOCCA implements generation of negative training data, and additionally a mode that requires only that the user specifies positives, motifs and a genome. MOCCA is licensed under the MIT license and is available on Github at https://github.com/bjornbredesen/MOCCA.

**Supplementary Information:**

The online version contains supplementary material available at 10.1186/s12859-021-04143-2.

## Background

*Cis*-regulatory elements (CREs) are DNA sequences that regulate gene expression [1]. CREs are enriched in sequence motifs, and a common task in genome analysis is the identification of CREs using machine learning models of their motif composition. There are many ways in which such machine learning models can be constructed, depending on the selection of motifs, the machine learning method of choice and the feature space formulation used. Log-odds models such as the PREdictor [2] model motif occurrence frequencies by weighting each feature with the logarithm of occurrence frequencies in positive versus negative training examples. Ringrose et al. [2] found that motif pairs are predictive of Polycomb Response Elements (PREs)—a class of CREs that maintain epigenetic memory—whereas singular motifs are not.

The method of Support Vector Machines (SVMs) solves a binary classification problem in a given feature space by placing a hyperplane such that the margin from the hyperplane to opposing classes is maximized [3]. A soft margin enables treating a subset of observations as noise, reducing over-fitting. SVMs can solve non-linear modelling problems by use of kernel functions that map an input feature space to a higher-dimensional space, and can solve multi-class classification problems by constructing one classifier per class boundary. Zeng et al. [4] trained SVMs to model PRE sequences in terms of their singular motif occurrence frequencies and found that models with non-linear kernels yield superior generalization to linear kernels. SVM-MOCCA [5] models the local motif occurrence and dinucleotide landscape around motif occurrences using one SVM per motif, in order to predict whether motif occurrences are similar to those within CREs, and combines the resulting SVMs with a log-odds model. In the prediction of *Drosophila* PREs, SVM-MOCCA displayed superior generalization performance when compared to other methods [5]. SVMs have also been used for the modelling of Polycomb targeting in vertebrate genomes [6, 7], for the prediction of mammalian enhancers [8], and also for other bioinformatics prediction problems [9].

A Random Forest (RF) [10] is an ensemble of decision trees trained with randomization, such as by the random selection of training data per tree, by the random sampling of the feature space, or by the random selection of splits at each node. Predictions are combined, for example by voting or averaging. Importantly, the randomizations that RFs employ reduce overfitting. Two studies have modelled Boundary Elements (BEs) with Random Forests: one study using 4-mer occurrence frequencies [11] and the other using occurrence frequencies of DNA-binding motifs of known BE-interacting factors [12]. RFs have also been used in other bioinformatics prediction problems [13, 14].

With [5], we published a novel hierarchical modelling method—the Support Vector Machine Motif Occurrence Combinatorics Classification Algorithm (SVM-MOCCA)—, which substantially improves generalization to PREs compared with previous methods. However, we did not publish a software package, which would be useful for researchers wishing to apply SVM-MOCCA to new problems. We had not tested how SVM-MOCCA would generalize if Position Weight Matrix (PWM) motifs were employed. Such a package could also include functionality that would simplify use. Additionally, the questions remained open of how well a derivative MOCCA model using a different machine learning method would perform, and of how well MOCCA-based methods would perform at a new modelling task.

Here we present MOCCA—Motif Occurrence Combinatorics Classification Algorithms—, a suite for modelling regulatory DNA sequences in terms of motif composition. MOCCA implements a variety of model and feature space formulations that can be used on their own or combined into new modelling approaches. MOCCA provides an efficient implementation of SVM-MOCCA and also a new CRE model—the Random Forest Motif Occurrence Combinatorics Classification Algorithm (RF-MOCCA). MOCCA supports use of both IUPAC and PWM motifs and implements functionality that facilitates ease of use. MOCCA is open source and extensible.

## Implementation

MOCCA builds predictive models of CREs based on user-specified motifs, training sequences and model specifications. After building/training the model, several optional steps can be executed: (1) the application to validation sequences and the calculation of validation statistics, (2) the calibration of a prediction threshold for a desired expected precision and (3) genome-wide prediction.

Sequences are supplied to MOCCA in FASTA format or can be generated by either an i.i.d. model or an *N*-th order Markov chain. Sequences are specified by the user as training sequences, validation sequences or calibration sequences.

Two types of motifs are supported: IUPAC nucleotide code [15] motifs and Position Weight Matrix (PWM) motifs. IUPAC nucleotide code motifs can be specified either individually as command-line arguments or together in an XML file. For efficient parsing of IUPAC motif occurrences, MOCCA constructs a Finite State Machine that parses occurrences of all IUPAC motifs in parallel. For PWM motifs, the file format used by the FlyFactorSurvey [16] for horizontal Position Specific Scoring Matrices (PSSMs) is supported. Example motif XML files and PWM files are included with MOCCA. For each PWM motif, a motif occurrence prediction threshold is set, in order to define discrete motif occurrences. The PWM threshold can be specified by the user or can be calibrated by MOCCA for an expected number of occurrences per kilobase using an i.i.d. model.

The models implemented in MOCCA are listed in Table 1. MOCCA implements three classes of models that have previously been applied for the prediction of Polycomb/Trithorax Response Elements: (1) the Dummy PREdictor [5], (2) the CPREdictor [2, 5] and (3) SVM-MOCCA [5]. In addition, MOCCA presents a new hierarchical model: the Random Forest Motif Occurrence Combinatorics Classification Algorithm (RF-MOCCA).

SVM-MOCCA and RF-MOCCA model CRE sequences by training one SVM/RF per motif, each of which models the local sequence landscape surrounding each occurrence of their respective motif within a 500bp window centred at the occurrence. The following local sequence features can be combined: motif occurrence frequencies, dinucleotide frequencies and GC content. Positive motif occurrence predictions are finally combined using a log-odds model for the prediction of whole regulatory elements. RF-MOCCA has the same model structure as SVM-MOCCA but with the SVMs replaced with Random Forests (RFs).

**Table 1:** Models implemented in MOCCA. * Requires optional integration with Shogun [17]

Model type	Description
Unweighted sum	Sum of specified feature spaces
Log-odds	Log-odds model of specified feature spaces
General SVM	SVM model of specified feature spaces
General RF	RF model of specified feature spaces
General LDA *	LDA model of specified feature spaces
General Averaged Perceptron *	Averaged Perceptron model of specified feature spaces
CPREdictor [5]	Re-implementation of the PREdictor [2] method
Dummy PREdictor	Unweighted version of the PREdictor
SVM-MOCCA [5]	Modelling sequence landscapes around motif occurrences using SVMs
RF-MOCCA	Modelling sequence landscapes around motif occurrences using RFs

Additionally, MOCCA supports constructing models based on a base model and desired feature space formulations. The supported base models are: dummy models (unweighted sums), log-odds models (as used in [2]), Support Vector Machines (SVMs) and Random Forests (RFs). In addition, the following base models are supported via an optional integration with the Shogun [17] machine learning library: Linear Discriminant Analysis (LDA) and Averaged Perceptron. In order to enable the Shogun integration, Shogun must be installed and MOCCA built with an additional flag, documented in the README for MOCCA on Github. Supported feature space formulations include motif occurrence frequencies (as used in [4]), motif pair occurrence frequencies (as used in [2]) and novel feature spaces that incorporate motif distancing.

To aid in precise candidate CRE prediction, MOCCA implements core-CRE prediction for SVM-MOCCA and RF-MOCCA. Core-CRE prediction enables the application of SVM-MOCCA and RF-MOCCA using large sliding window and step sizes, and subsequently the prediction of shorter CRE cores within larger predictions. For each prediction, the algorithm applies each SVM/RF to classify motif occurrences, and then scores every non-redundant sub-window using the log-odds model. The non-redundant sub-windows are defined as every window delimited by a pair of motif occurrences or centred at a single occurrence, with 250bp added to each end to account for the feature window size used by each SVM/RF. The sub-window with the highest score per base pair is predicted as a core CRE.

To aid in ease of use, MOCCA additionally implements a mode that requires only that the user specifies a set of motifs, positive training sequences and a genome sequence. MOCCA then divides the positives into training and test portions and constructs negative training, test and calibration data. MOCCA finally calibrates a prediction threshold and predicts candidate CREs genome-wide.

MOCCA has been implemented in C++. MOCCA has a minimal number of dependencies, which are included with the source distribution, simplifying installation. For the SVM implementation, MOCCA links with libsvm [18]. For the RF implementation, MOCCA links with Ranger [19]. The Ranger library supports multi-processing, in turn adding multi-processing capability to the general RF model and to RF-MOCCA. Other models in MOCCA are currently limited to the use of a single core. MOCCA also optionally links with Shogun [17]. XML parsing is enabled by the RapidXML library [20]. MOCCA is licensed under the MIT license, and included libraries (libsvm, Ranger and RapidXML) are under compatible licenses. The source code of MOCCA is included in Additional file 1.

## Results and discussion

We have previously applied the Dummy PREdictor, the CPREdictor and SVM-MOCCA in the modelling of *D. melanogaster* PREs, and we found that SVM-MOCCA yielded superior generalization performance. For the suite MOCCA presented here, we developed a new CRE model: the Random Forest Motif Occurrence Combinatorics Classification Algorithm (RF-MOCCA). Additionally, we added support for Position Weight Matrix (PWM) motifs. We were interested in how the generalization performance of RF-MOCCA compares with that of the previously tested models and in how the use of PWM motifs may influence model generalization. We were also interested in how our methods would fare with modelling a new class of CREs.

In order to compare model generalization performance, we applied the aforementioned models with training and test data comparable to the set that we used in [5], with Schwartz et al. [21] PREs as positives versus three classes of negatives: dummy genomic, dummy PREs and coding sequences, using 110 randomly selected sequences of each class for training, and 50 independent positives and 5000 negatives for validation (as described in [5]). In order to assess generalization performance independently of random variation, the procedure was repeated 20 times, and means and $95\%$ confidence intervals were calculated. SVM-MOCCA and RF-MOCCA are multi-class models, and we trained SVM-MOCCA and RF-MOCCA using PREs and all three classes of non-PREs. These three negative sets reflect the heterogeneity of non-PREs more precisely than any single set. CPREdictor is a two-class model, and we trained it with PREs and dummy PREs (as we did previously in [5]). For the features of SVM-/RF-MOCCA, we used local motif and dinucleotide occurrence frequencies. We used two motif sets for PREs: the same set that we used in [5], including the GTGT motif, noted as M2019, and a set composed mainly of PWM motifs, noted as MPWM. The MPWM set consists of PWM motifs for Pho, Zeste and GAF, acquired from the Fly Factor Survey [16], with a threshold calibrated for an expected occurrence frequency of one per kilobase. For details on data preparation and experiments, see Additional file 2.

To test MOCCA on a new CRE modelling task, we trained and applied our MOCCA models to boundaries of Topologically Associating Domains (TADs) extracted from the Sexton et al. [22] study, using an identical cross-validation scheme to that used with PREs and using motifs from a previous insulator/Boundary Element (BE) predictor, cdBEST [23], noted as M2012. For comparison, we applied cdBEST [23] to the same data. To see if we could further improve generalization, we tested a second motif set with motifs added for the more recently discovered insulator binding factors Ibf1/2 [12, 24], noted as M2020.

Finally, we applied all methods for genome-wide prediction (Additional files 4–5) and analysed the fractions of predictions in accessible chromatin that overlap with relevant signals from modENCODE or with experimentally determined PREs or BEs. We additionally predicted core CREs (Additional files 6–7).

**Fig. 1:**
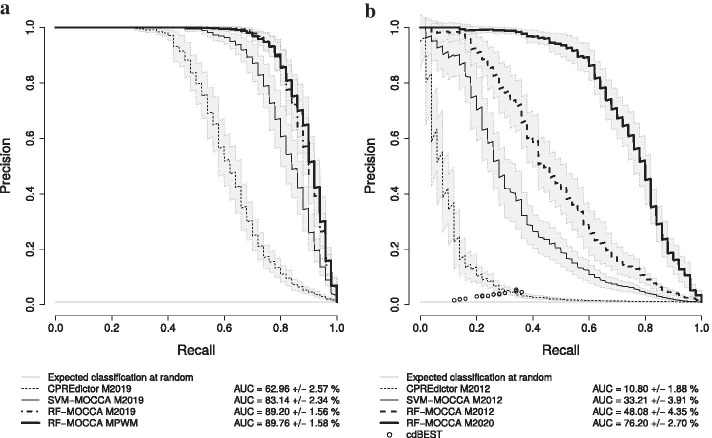
RF-MOCCA improves separation of both PREs and TAD boundaries from background. Cross-validation Precision/Recall curves with 110 positives and negatives for training, and 50 positives and 5000 negatives for testing, as in [5]. **a** Generalization to PREs [21] versus dummy genomic sequences. **b** Generalization to TAD boundaries [22] versus dummy genomic sequences

Of the methods tested, RF-MOCCA yielded the best generalization performance to both PREs and BEs, with a 1.07-fold increase in the area under the Precision/Recall curve (PRC AUC) over that of SVM-MOCCA for PREs (Fig. 1, panel A) and a 1.45-fold increase for BEs (Fig. 1, panel B). The use of a smaller set of PWM motifs for PREs (MPWM) yielded a generalization comparable to that of the set of IUPAC motifs that we used in [5] (M2019) (Fig. 1, panel A). For BEs, SVM-/RF-MOCCA yield a 3.07–4.45-fold improvement in generalization over that of the CPREdictor (Fig. 1, panel B). Also for BEs, adding the Ibf1/2 motifs (M2020) yielded a further 1.58-fold increase. The improvement in generalization is larger for the introduction of the new motifs than it is for a change from SVM-MOCCA to RF-MOCCA. Although we added these motifs for RF-MOCCA only, their addition can be expected to similarly improve the generalization of other models, such as SVM-MOCCA and the CPREdictor. cdBEST [23] yielded comparably poor performance (Fig. 1, panel B). As cdBEST makes binary predictions for each sequence, there is one point per experimental repeat for cdBEST.

For comparison, we also modelled PREs and BEs using general SVMs and RFs in terms of occurrence frequencies of the same motifs and of a comprehensive set of 4-mers, which yielded comparatively poor generalization (see Additional file 3: Fig. S1). The general SVM trained here to model PREs using motif occurrence frequencies is similar to the SVM used by Zeng et al. [4], the EpiPredictor, as both are trained with PREs and use motif occurrence frequencies as features—differences include that Zeng et al. [4] filtered predictions by GC content and scored by the total number of motif occurrences, rather than using the SVM decision function. The RF trained with 4-mers to model BEs is similar to the model used by Bednarz et al. [11] with sequence features only.

**Fig. 2:**
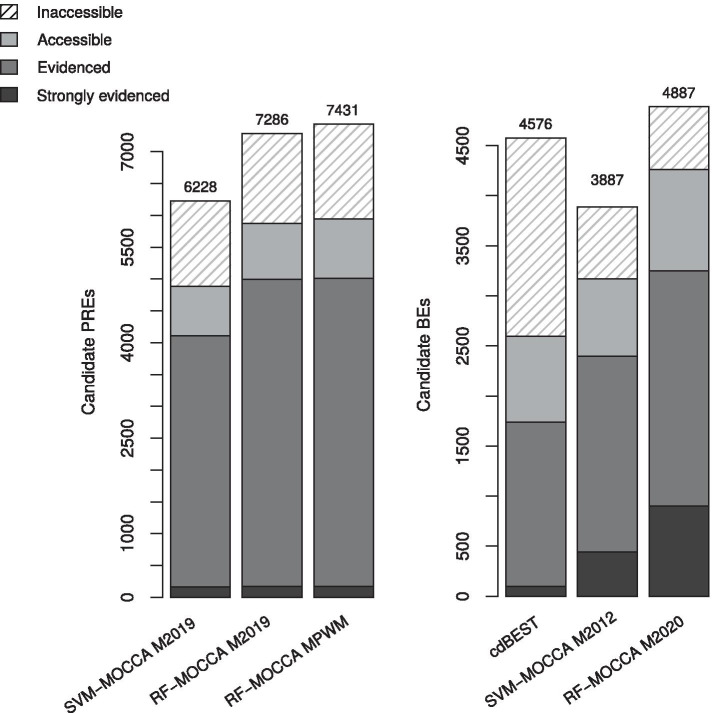
RF-MOCCA predicts more PcG-enriched candidate PREs and boundary element factor enriched candidate BEs. The numbers of candidate PREs and BEs predicted genome-wide by different models. Predictions in accessible chromatin are broken down into “strongly evidenced” (PRE predictions that overlap with PREs from [26] and BE predictions that overlap with TAD boundaries from [22]), “evidenced” (PRE predictions that overlap with modENCODE signals [27] of Pc, Psc or dSFMBT and BE predictions that overlap with modENCODE signals [27] of BEAF-32 or CP190) and “accessible” (the remainder)

We performed a second cross-validation for BEs, with boundaries deduced from data from Ramirez et al. [12], which yielded a similar trend to that observed with the Sexton et al. [22] data, with overall lower PRC AUCs (see Additional file 3: Fig. S2). SVM-MOCCA and RF-MOCCA yield comprehensive sets of genome-wide predictions of candidate PREs and BEs (see Additional files 4–7) that are highly enriched in relevant experimental signals (Fig. 2).

**Table 2:** Running times for genome-wide prediction in *D. melanogaster*, using the same training data as for the first cross-validation iteration, on an Intel Core i9-9900K CPU (3.6GHz, 8 cores)

Model type	Running time (hh:mm:ss)	Cores used
jPREdictor M2019	0:01:21	1
CPREdictor M2019 (PREs)	0:00:06	1
SVM-MOCCA M2019 (PREs)	8:20:05	1
RF-MOCCA M2019 (PREs)	14:01:48	8
cdBEST	9:42:23	1

Running times for genome-wide prediction are listed in Table 2. MOCCA’s implementation of the PREdictor algorithm is significantly faster than the jPREdictor [25] for the same configuration, taking only $7 \%$ of the time for a genome-wide prediction. The decreased time taken can be attributed to the implementation of motif occurrence parsing and handling, where MOCCA constructs a Finite State Machine and parses occurrences in time linear of sequence length. SVM-MOCCA takes substantially longer (5000-fold), which can be attributed to applying a non-linear multi-class SVM for every occurrence of every motif. The running time can be tuned by choosing a different kernel (such as linear). The Ranger library supports multi-core processing, and we employed eight cores for our benchmark. RF-MOCCA still takes the longest, which can similarly be attributed to applying a large number of trees to every occurrence of every motif. The running time of RF-MOCCA can be tuned by adjusting the number of trees and tree depth, both of which can be specified with arguments to MOCCA. Finally, the running times of SVM-/RF-MOCCA can be shortened by reducing the number of motifs or sequence classes or by using a larger sliding window size and larger step size for genome-wide prediction.

There are multiple tradeoffs to consider when choosing between SVM-MOCCA and RF-MOCCA for new modelling problems. Firstly, while RF-MOCCA yielded superior generalization over SVM-MOCCA with a quadratic kernel, RF-MOCCA required longer processing time. Secondly, SVM-MOCCA lends itself more readily to model interpretation than does RF-MOCCA, as a linear or quadratic SVM kernel can be reformulated as feature or feature pair weights, respectively, which we previously did in order to analyse an SVM-MOCCA model [5].

In summary, RF-MOCCA further improves generalization to independent PREs over the already excellent generalization observed with SVM-MOCCA. SVM-MOCCA and RF-MOCCA also successfully distinguish instances of a second class of CREs, Boundary Elements, and outperform previous methods. Notably, SVM-MOCCA and RF-MOCCA both yield superior generalization to traditional 4-spectrum and motif occurrence frequency SVMs and RFs. The MOCCA suite provides an efficient implementation of both methods. The use of PWM motifs yielded a generalization comparable to the use of IUPAC motifs, and the application of SVM-MOCCA and RF-MOCCA to new modelling problems is simplified by the wide availability of high-quality PWMs for a number of DNA-binding factors in a number of organisms.

## Conclusions

MOCCA is a flexible suite for the modelling of regulatory DNA sequences in terms of motif composition. It provides a variety of motif-based machine learning methods for the task and functions that simplify the process of model training and genome-wide prediction, including the generation of negative sequences and prediction threshold calibration, and also a mode that requires only that the user specifies motifs, positive training examples and a genome sequence. We have previously published the Support Vector Machine Motif Occurrence Combinatorics Classification Algorithm (SVM-MOCCA), which we found to yield several-fold improvements in generalization to independent PREs over previously published methods [5]. MOCCA moves beyond our work in [5] and not only presents an efficient, configurable and polished implementation of SVM-MOCCA but also a new CRE modelling method—the Random Forest Motif Occurrence Combinatorics Classification Algorithm (RF-MOCCA)—, which further improves upon the generalization of SVM-MOCCA. MOCCA additionally adds support for PWM motifs and implements functionality that facilitates ease of use. We applied SVM-MOCCA and RF-MOCCA to the problems of modelling PREs and Boundary Elements (BEs)/TAD boundaries, making this the first study to model PREs using RF-based methods and also the first to model BEs using MOCCA-based methods. SVM-MOCCA and RF-MOCCA improve generalization to both PREs and BEs over that of generic SVMs and RFs, with RF-MOCCA yielding the best generalization in both cases (see Additional file 3: Fig. S1). Accordingly, we have demonstrated that our methods generalize well to new modelling problems and are potentially useful for a number of additional CRE modelling tasks and CRE modelling in other organisms.

Additionally, MOCCA supports the training and application of log-odds, general Support Vector Machine and general Random Forest models, allowing the user to mix and match feature spaces with his or her motifs of choice and to explore potentially novel ways of modelling the motif occurrence landscapes of sequences of interest. MOCCA is open source and extensible.

## Availability and requirements

- **Project name:** Motif Occurrence Combinatorics Classification Algorithms (MOCCA)
- **Project home page:** https://github.com/bjornbredesen/MOCCA/
- **Operating system(s):** UNIX-based systems
- **Programming language:** C++
- **License:** MIT license
- **Any restrictions to use by non-academics:** None

## Supplementary Information

**Additional file 1**. MOCCA source code.

**Additional file 2**. Supplementary methods. Details of data acquisition and processing, and experiments and analyses.

**Additional file 3**. Supplementary figures.

**Additional file 4**. Candidate PREs predicted by RF-MOCCA M2019.

**Additional file 5**. Candidate BEs predicted by RF-MOCCA M2020.

**Additional file 6**. Core PREs predicted by RF-MOCCA M2019.

**Additional file 7**. Core BEs predicted by RF-MOCCA M2020.

## Data Availability

The genome-wide predictions generated in this study are available in Additional files 4–7.

## Abbreviations

CRE: *Cis*-regulatory element
BE: Boundary Element
i.i.d.: independent and identically distributed
LDA: Linear discriminant analysis
MOCCA: Motif occurrence combinatorics classification algorithms
PRE: Polycomb/trithorax response element
PWM: Position weight matrix
PSSM: Position specific scoring matrix
RF: Random forest
SVM: Support vector machine
TAD: Topologically associating domain
