# Supplementary material for: MOCCA: a flexible suite for modelling DNA sequence motif occurrence combinatorics
Source: BMC Bioinformatics. 2021 May 7;22:234. doi: 10.1186/s12859-021-04143-2 (PMC8105988; doi:10.1186/s12859-021-04143-2)
Supplement: Supplementary file 2 — Additional file 2. Supplementary methods. Details of data acquisition and processing, and experiments and analyses. [file 12859_2021_4143_MOESM2_ESM.pdf]

Supplementary methods

Genome assemply

We used the *D. melanogaster* genome assembly release 5 [1] for all analyses in the present work.

Polycomb/Trithorax Response Elements (PREs)

For comparability with our previous work [2], we acquired PcG-targets from Schwartz *et al.* [3], from the article's Supplementary Table S6, and converted coordinates from *D. melanogaster* genome assembly 4 to 5.

Boundary Elements (BEs)

As candidates for Boundary Elements (BEs), we downloaded Topologically Associating Domains (TADs) from Sexton *et al.* [4], from the article's Supplementary Table S1, and we placed 3kb-long regions centered at the boundaries between TADs.

For the second experiment with modelling BEs, we acquired TADs from the Ramirez *et al.* [5] study, from their Supplementary Data 1, and we placed 3kb-long regions centered at the boundaries between TADs.

Markov chains

For generating negatives, we used 4th-order Markov chains. We used the Markov chain implementation from Gnocis (article submitted), with pseudocounts of 1. We trained a Markov chain genome-wide and generated 3kb-long sequences, henceforth called *dummy genomic sequences*. We also trained Markov chains on both positives (PREs or BEs) and generated 3kb-long sequences, henceforth called *dummy PREs* or *dummy BEs*, depending on the training class.

Coding sequences

As a third class of negatives, we acquired annotated coding sequences from FlyBase [1]. We concatenated the coding sequences and split them into non-overlapping 3kb-long fragments.

ModENCODE Polycomb targets

We downloaded PcG-enriched regions from modENCODE and denoted predictions that overlap with one or more relevant modENCODE regions as "evidenced".

Base path: ftp://data.modencode.org/D.melanogaster

- Non-TF-Chromatin-binding-factor/ChIP-seq/computed-peaks_gff3/Pc:Developmental-Stage=Embryos-14-16-hr-OR#Strain=Oregon-R:ChIP-seq:Rep-1::Dmel_r5.32:modENCODE_3957:816.gff3.gz
- Non-TF-Chromatin-binding-factor/ChIP-seq/computed-peaks_gff3/Pc:Developmental-Stage=Embryos-14-16-hr-OR#Strain=Oregon-R:ChIP-seq:Rep-1::Dmel_r5.32:modENCODE_3957:948.gff3.gz
- Non-TF-Chromatin-binding-factor/ChIP-seq/computed-peaks_gff3/Psc:Developmental-Stage=Embryos-14-16-hr-OR#Strain=Oregon-R:ChIP-seq:Rep-1::Dmel_r5.32:modENCODE_3960:1817.gff3.gz
- Non-TF-Chromatin-binding-factor/ChIP-seq/computed-peaks_gff3/Psc:Developmental-Stage=Embryos-14-16-hr-OR#Strain=Oregon-R:ChIP-seq:Rep-1::Dmel_r5.32:modENCODE_3960:1949.gff3.gz
- Non-TF-Chromatin-binding-factor/ChIP-seq/computed-peaks_gff3/Psc:Developmental-Stage=Embryos-14-16-hr-OR#Strain=Oregon-R:ChIP-seq:Rep-1::Dmel_r5.32:modENCODE_3960:repset.17400034.gff3.gz
- Non-TF-Chromatin-binding-factor/ChIP-seq/computed-peaks_gff3/dSFMBT:Developmental-Stage=Larvae-3rd-instar#Strain=Oregon R:ChIP-seq:Rep-1::Dmel_r5.32:modENCODE_5072:2425.gff3.gz
- Non-TF-Chromatin-binding-factor/ChIP-seq/computed-peaks_gff3/dSFMBT:Developmental-Stage=Larvae-3rd-instar#Strain=Oregon R:ChIP-seq:Rep-1::Dmel_r5.32:modENCODE_5072:2426.gff3.gz
- Non-TF-Chromatin-binding-factor/ChIP-seq/computed-peaks_gff3/dSFMBT:Developmental-Stage=Larvae-3rd-instar#Strain=Oregon R:ChIP-seq:Rep-1::Dmel_r5.32:modENCODE_5072:repset.18482408.gff3.gz

- Non-TF-Chromatin-binding-factor/ChIP-seq/computed-peaks_gff3/dSFMBT:Developmental-Stage=Mixed-Adult#Strain=Oregon-R#Tissue=Heads-OR#organism-part=Tissue-Heads-OR-GK-1:ChIP-seq:Rep-1::Dmel_r5.32:modENCODE_5577:2427.gff3.gz
- Non-TF-Chromatin-binding-factor/ChIP-seq/computed-peaks_gff3/dSFMBT:Developmental-Stage=Mixed-Adult#Strain=Oregon-R#Tissue=Heads-OR#organism-part=Tissue-Heads-OR-GK-1:ChIP-seq:Rep-1::Dmel_r5.32:modENCODE_5577:2428.gff3.gz
- Non-TF-Chromatin-binding-factor/ChIP-seq/computed-peaks_gff3/dSFMBT:Developmental-Stage=Mixed-Adult#Strain=Oregon-R#Tissue=Heads-OR#organism-part=Tissue-Heads-OR-GK-1:ChIP-seq:Rep-1::Dmel_r5.32:modENCODE_5577:repset.18972728.gff[illegible]

ModENCODE Boundary Elements

We downloaded regions for boundary element–associated factors from modENCODE and denoted predictions that overlap with one or more relevant modENCODE regions as “evidenced”.

Base path: ftp://data.modencode.org/D.melanogaster

- Non-TF-Chromatin-binding-factor/ChIP-seq/computed-peaks_gff3/CP190:Developmental-Stage=Embryos-14-16-hr-OR#Strain=Oreg[illegible] R:ChIP-seq:Rep-1::Dmel_r5.32:modENCODE_3959:1963.gff3.gz
- Non-TF-Chromatin-binding-factor/ChIP-seq/computed-peaks_gff3/CP190:Developmental-Stage=Embryos-14-16-hr-OR#Strain=Oreg[illegible] R:ChIP-seq:Rep-1::Dmel_r5.32:modENCODE_3959:1971.gff3.gz
- Non-TF-Chromatin-binding-factor/ChIP-seq/computed-peaks_gff3/CP190:Developmental-Stage=Embryos-14-16-hr-OR#Strain=Oreg[illegible] R:ChIP-seq:Rep-1::Dmel_r5.32:modENCODE_3959:repset.17400046.gff3.gz
- Non-TF-Chromatin-binding-factor/ChIP-seq/computed-peaks_gff3/BEAF32A-and-BEAF42B:Developmental-Stage=Embryos-14-16-hr-OR#Strain=Oregon-R:ChIP-seq:Rep-1::Dmel_r5.32:modENCODE_3954:1964.gff3.gz
- Non-TF-Chromatin-binding-factor/ChIP-seq/computed-peaks_gff3/BEAF32A-and-BEAF42B:Developmental-Stage=Embryos-14-16-hr-OR#Strain=Oregon-R:ChIP-seq:Rep-1::Dmel_r5.32:modENCODE_3954:1972.gff3.gz
- Non-TF-Chromatin-binding-factor/ChIP-seq/computed-peaks_gff3/BEAF32A-and-BEAF42B:Developmental-Stage=Embryos-14-16-hr-OR#Strain=Oregon-R:ChIP-seq:Rep-1::Dmel_r5.32:modENCODE_3954:repset.17400048.gff3.gz

DNA accessibility

In order to assess the chromatin accessibility of predictions, we identified overlaps with DNaseI-seq peaks. We downloaded DNaseI-seq peaks from the Berkeley Drosophila Transcription Network Project (BDTNP) (http://bdtnp.lbl.gov:8080/Fly-Net/access.jsp) for embryonic stages 5, 9, 10, 11 and 14. Coordinates were converted from Dm2 to *D. melanogaster* genome assembly release 5 (Dm3) using CrossMap [6], using a LiftOver map from UCSC [7].

Polycomb Response Element DNA sequence motifs

The M2019 motif set is identical to the one used in [2].

Polycomb/Trithorax Response Elements – M2019

Name	Motif	Mismatches allowed
EN 1	`GSNMACGCCCC`	1
G10	`GAGAGAGAGA`	1
GAF	`GAGAG`	0
PF	`GCCATHWY`	0
PM	`CNGCCATNDNND`	0
PS	`GCCAT`	0
Z	`YGAGYG`	0
GT	`GTGT`	0

For the MPWM motif set, we replaced the Pho, Zeste- and GAF motifs with Position Specific Scoring Matrices (PSSMs) from the Fly Factor Survey [8].

Polycomb/Trithorax Response Elements – MPWM

Name	Motif	Mismatches allowed
EN 1	`GSNMACGCCCC`	1
GAF	Fly Factor Survey PSSM, FlyReg	N/A
Pho	Fly Factor Survey PSSM, SOLEXA	N/A
Zeste	Fly Factor Survey PSSM, FlyReg	N/A
GT	`GTGT`	0

Boundary Element DNA sequence motifs

The M2012 motif set is as was used in [9].

Boundary Elements – M2012

Name	Motif	Mismatches allowed
BEAF	`CGATA`	0
Zw5	`GCTGMG`	0
GAF	`GAGAG`	0
Su(Hw)-M1	`YRYTGCATAYYY`	0
Su(Hw)-M2	`YWGCMTACTTHY`	0
Elba	`MCAATAAG`	0
CTCF-M1	`MHRGRKGKCGCY`	0
CTCF-M2	`YAGRKGKCGC`	0
CTCF-M3	`RRCGCCMYCYRKY`	0
Fab-7Motif	`CCAATTGG`	0

For M2020, we replaced the Su(Hw) motifs with a Position Specific Scoring Matrix from the Fly Factor Survey [8]. Additionally, we acquired peaks for Ibf1 and Ibf2 from [10], extracted the underlying sequences, performed motif discovery using MEME-ChIP [11] and added the top three motifs for each factor (all of which were IUPAC motifs discovered by DREME [12]) to our list.

Boundary Elements – M2020

Name	Motif	Mismatches allowed
BEAF	`CGATA`	0
Zw5	`GCTGMG`	0
GAF	`GAGAG`	0
Su(Hw)	Fly Factor Survey PSSM, FlyReg	N/A
Elba	`MCAATAAG`	0
CTCF-M1	`MHRGRKGKCGCY`	0
CTCF-M2	`YAGRKGKCGC`	0
CTCF-M3	`RRCGCCMYCYRKY`	0
Fab-7Motif	`CCAATTGG`	0
Ibf1-M1	`RTGTARA`	0
Ibf1-M2	`AGRTGKC`	0
Ibf1-M3	`ATTYTAC`	0
Ibf2-M1	`HGTARAA`	0
Ibf2-M2	`AGRTGKCG`	0
Ibf2-M3	`ATGTMRA`	0

Cross-validation

In order to aid comparability with our previous work [2], we used an identical cross-validation procedure, for both PREs and BEs. Models were trained with 110 positive and negative training sequences (110 for each negative class for SVM-MOCCA and RF-MOCCA) and applied to score 50 independent positives and 5000 independent negatives. In order to account for random variability, we repeated the procedure 20 times.

Model configurations

For the CPREdictor, we used a window size of 500bp, and for SVM-MOCCA and RF-MOCCA a window size of 3kb, as in [2]. For MOCCA models, we used a step size of 100bp. For genome-wide prediction of BEs with RF-MOCCA, in order to reduce running time, we used an increased step size of 1000bp. For SVM-MOCCA, a quadratic kernel (polynomial degree 2), was used. For RF-MOCCA, 500 trees were used. For the features of SVM-MOCCA and RF-MOCCA, we used local motif and dinucleotide occurrence frequencies. For core-CRE prediction, we used a step size of 1000 and the default mode (continuous maximum).

When applying the jPREdictor, to aid comparability, we used the M2019 motifs, a window size of 500, and step size of 100.

When applying cdBEST, we used the basic-version script. In our cross-validation procedure, we applied cdBEST to each of our test sequences separately, and checked whether cdBEST had predicted any boundaries in the sequence, by parsing the output file “hits_table.txt”, yielding a binary prediction per sequence. Based on these predictions, we calculated 20 confusion matrices (one per cross-validation fold) per test case (BEs versus dummy genomic sequences, and BEs versus dummy BEs).

References

[1] S. J. Marygold, P. C. Leyland, R. L. Seal, J. L. Goodman, J. Thurmond, V. B. Strelets, R. J. Wilson, and F. Consortium, “FlyBase: improvements to the bibliography,” *Nucleic Acids Res*, vol. 41, no. D1, pp. D751–7, 2012.

[2] B. A. Bredesen and M. Rehmsmeier, “DNA sequence models of genome-wide Drosophila melanogaster Polycomb binding sites improve generalization to independent Polycomb Response Elements,” *Nucleic Acids Res*, vol. 47, no. 15, pp. 7781–97, 2019.

[3] Y. B. Schwartz, T. G. Kahn, P. Stenberg, K. Ohno, R. Bourgon, and V. Pirrotta, “Alternative epigenetic chromatin states of polycomb target genes,” *PLoS Genet*, vol. 6, no. 1, p. e1000805, 2010.

[4] T. Sexton, E. Yaffe, E. Kenigsberg, F. Bantignies, B. Leblanc, M. Hoichman, H. Parrinello, A. Tanay, and G. Cavalli, “Three-dimensional folding and functional organization principles of the Drosophila genome,” *Cell*, vol. 148, no. 3, pp. 458–72, 2012.

[5] F. Ramírez, V. Bhardwaj, L. Arrigoni, K. C. Lam, B. A. Grüning, J. Villaveces, B. Habermann, A. Akhtar, and T. Manke, “High-resolution TADs reveal DNA sequences underlying genome organization in flies,” *Nat Commun*, vol. 9, no. 1, pp. 1–15, 2018.

[6] H. Zhao, Z. Sun, J. Wang, H. Huang, J.-P. Kocher, and L. Wang, “CrossMap: a versatile tool for coordinate conversion between genome assemblies,” *Bioinformatics*, vol. 30, no. 7, pp. 1006–7, 2014.

[7] W. J. Kent, C. W. Sugnet, T. S. Furey, K. M. Roskin, T. H. Pringle, A. M. Zahler, and D. Haussler, “The human genome browser at UCSC,” *Genome Res*, vol. 12, no. 6, pp. 996–1006, 2002.

[8] L. J. Zhu, R. G. Christensen, M. Kazemian, C. J. Hull, M. S. Enuameh, M. D. Basciotta, J. A. Brasefield, C. Zhu, Y. Asriyan, D. S. Lapointe, *et al.*, “FlyFactorSurvey: a database of Drosophila transcription factor binding specificities determined using the bacterial one-hybrid system,” *Nucleic Acids Res*, vol. 39, no. Database issue, pp. D111–7, 2011.

[9] A. Srinivasan and R. K. Mishra, “Chromatin domain boundary element search tool for Drosophila,” *Nucleic Acids Res*, vol. 40, no. 10, pp. 4385–95, 2012.

[10] S. Cuartero, U. Fresán, O. Reina, E. Planet, and M. L. Espinàs, “Ibf1 and Ibf2 are novel CP190-interacting proteins required for insulator function,” *EMBO J*, vol. 33, no. 6, pp. 637–47, 2014.

[11] P. Machanick and T. L. Bailey, “MEME-ChIP: motif analysis of large DNA datasets,” *Bioinformatics*, vol. 27, no. 12, pp. 1696–7, 2011.

[12] T. L. Bailey, “DREME: motif discovery in transcription factor ChIP-seq data,” *Bioinformatics*, vol. 27, no. 12, pp. 1653–9, 2011.
