# Supplementary material for: MOCCA: a flexible suite for modelling DNA sequence motif occurrence combinatorics
Source: BMC Bioinformatics. 2021 May 7;22:234. doi: 10.1186/s12859-021-04143-2 (PMC8105988; doi:10.1186/s12859-021-04143-2)
Supplement: Supplementary file 3 — Additional file 3. Supplementary figures. [file 12859_2021_4143_MOESM3_ESM.pdf]

Supplementary Figures

Supplementary Figure S1 - SVM-MOCCA and RF-MOCCA improve generalization over that of an SVM or RF with motif occurrence frequencies as features

We compared SVM-MOCCA and RF-MOCCA with a general SVM and a general RF. We trained the SVM and RF with a window size of 3kb, using the general SVM and RF implementations available in MOCCA. The SVM was trained with a quadratic kernel, and the RF was trained with 500 trees. As features, we used the occurrence frequencies (the “nOcc” feature set), either of all 4-mers (“SVM/RF 4-mers”), or of the M2019 motifs for PREs (“SVM/RF M2019”), and M2020 motifs for BEs (“SVM/RF M2020”).

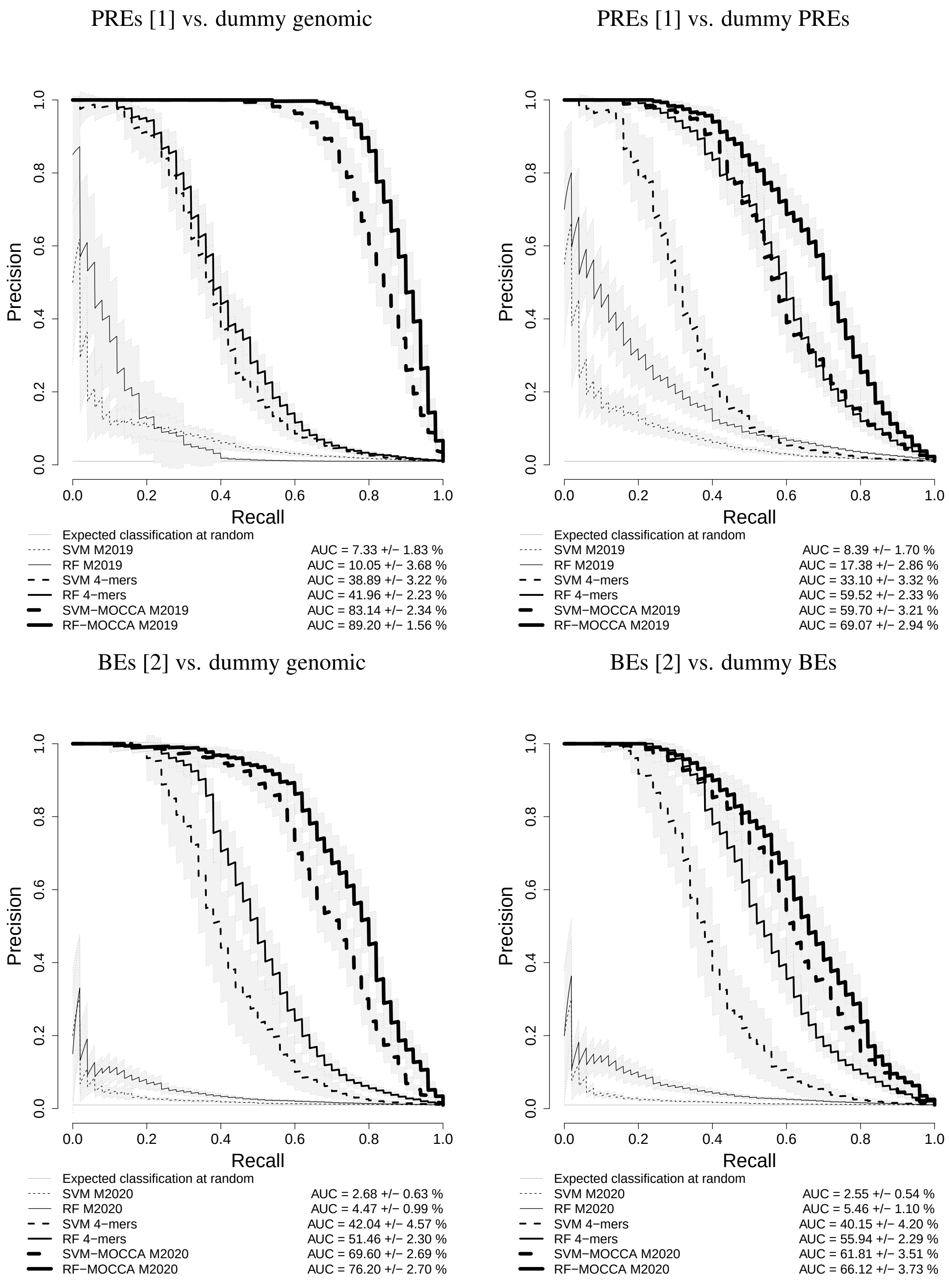

Supplementary Figure S2 - SVM-MOCCA and RF-MOCCA improve generalization to TAD boundaries from [3]

TAD boundaries [3] vs. dummy genomic

TAD boundaries [3] vs. dummy TAD boundaries

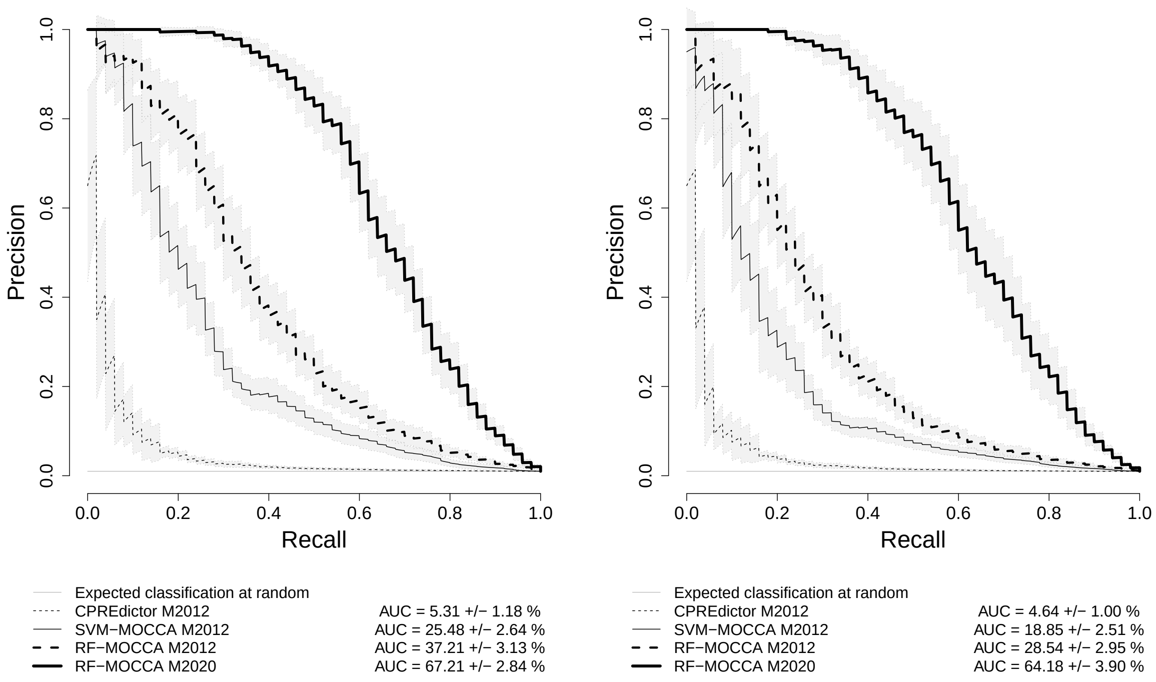

References

[1] Y. B. Schwartz, T. G. Kahn, P. Stenberg, K. Ohno, R. Bourgon, and V. Pirrotta, “Alternative epigenetic chromatin states of polycomb target genes,” *PLoS Genet*, vol. 6, no. 1, p. e1000805, 2010.

[2] T. Sexton, E. Yaffe, E. Kenigsberg, F. Bantignies, B. Leblanc, M. Hoichman, H. Parrinello, A. Tanay, and G. Cavalli, “Three-dimensional folding and functional organization principles of the Drosophila genome,” *Cell*, vol. 148, no. 3, pp. 458–72, 2012.

[3] F. Ramírez, V. Bhardwaj, L. Arrigoni, K. C. Lam, B. A. Grüning, J. Villaveces, B. Habermann, A. Akhtar, and T. Manke, “High-resolution TADs reveal DNA sequences underlying genome organization in flies,” *Nat Commun*, vol. 9, no. 1, pp. 1–15, 2018.
